# Retraction: Combined application of zinc and silicon alleviates terminal drought stress in wheat by triggering morpho-physiological and antioxidants defense mechanisms

**DOI:** 10.1371/journal.pone.0272195

**Published:** 2022-08-03

**Authors:** The PLOS ONE Editors

The *PLOS ONE* Editors retract this article [1] because it was identified as one of a series of submissions for which we have concerns about authorship, competing interests, and peer review. We regret that the issues were not addressed prior to the article’s publication.

AS, XW, TA, AS, MIjaz, SUA, MB, MAW, MC, SF, AQ, MJA, FA, and ATKZ did not agree with the retraction. MIrfan, SAA, MW, and KX either did not respond directly or could not be reached.
